# Cytoprotective Effects of *Gymnema inodorum* Against Oxidative Stress-Induced Human Dermal Fibroblasts Injury: A Potential Candidate for Anti-Aging Applications

**DOI:** 10.3390/antiox14091043

**Published:** 2025-08-24

**Authors:** Wattanased Jarisarapurin, Thanchanok Puksasook, Sarawut Kumphune, Nattanicha Chaiya, Pawinee Pongwan, Rawisada Pholsin, Issara Sramala, Satita Tapaneeyakorn

**Affiliations:** 1 National Nanotechnology Center (NANOTEC), National Science and Technology Development Agency (NSTDA), Khlong Luang District, Pathum Thani 12120, Thailand; thanchanok.puksasook@gmail.com (T.P.); nattanicha.cha@nanotec.or.th (N.C.); pawinee@nanotec.or.th (P.P.); rawisadap@gmail.com (R.P.); 2 Biomedical Engineering Institute, CMU-BIOPOLIS Building, Chiang Mai University, Mae-Hia District, Chiang Mai 50100, Thailand; wattanased.j@cmu.ac.th (W.J.); sarawut.kumphune@cmu.ac.th (S.K.); 3 Biomedical Engineering and Innovation Research Centre, CMU-BIOPOLIS Building, Chiang Mai University, Mae-Hia District, Chiang Mai 50100, Thailand; 4 Office of Research Administration, Chiang Mai University, Mae-Hia District, Chiang Mai 50100, Thailand

**Keywords:** phytochemical antioxidants, cutaneous fibroblasts, antioxidation, medicinal plant, Chiang Da vegetable

## Abstract

Repeated UV exposure, air pollution, and toxins promote skin oxidative stress. ROS destroy macromolecules, changing cellular mechanisms and signaling cascades. Inflammation and injury to skin cells degrade function and accelerate aging, causing wrinkles, firmness loss, and dermatological disorders. *Gymnema inodorum* (GI) contains phytochemical antioxidants such polyphenols and triterpenoids that lower ROS and strengthen skin. GI extracts (GIEs) have never been examined for their effects on dermal skin fibroblasts’ oxidative stress and intracellular cytoprotective mechanisms. In this study, GIEs were prepared as a water extract (GIE0) and ethanol extracts with concentrations ranging from 20% to 95% *v*/*v* (GIE20, GIE40, GIE60, GIE80, and GIE95). These extracts were assessed for phytochemical content, antioxidant capacity, and free radical scavenging efficacy. The results were compared to a commercially available native Gymnema extract (NGE) obtained from *Gymnema sylvestre*. During principal component analysis (PCA), the most effective extracts were identified and subsequently evaluated for their ability to mitigate oxidative stress in fibroblasts. Cytoprotective effects of GIE and NGE against H_2_O_2_-induced human dermal fibroblast injury were investigated by cell viability, intracellular ROS production, and signaling pathways. GIE0, GIE80, GIE95, and NGE were the best antioxidants. By preserving ROS balance and redox homeostasis, GIE and NGE reduce fibroblast inflammation and oxidative stress-induced damage. Decreased ROS levels reduce MAPK/AP-1/NF-κB and PI3K/AKT/NF-κB signaling pathways, diminishing inflammatory cytokines. In conclusion, GIE and NGE have antioxidant and anti-inflammatory capabilities that can reduce H_2_O_2_-induced fibroblast oxidative stress and damage, thereby preventing skin aging and targeting cancer-associated fibroblasts.

## 1. Introduction

Amidst climate change, environmental impacts are becoming more prevalent and detrimental, resulting in substantial health hazards. The skin, our primary defense against external threats, is presented with various challenges. Long-term exposure to these extrinsic stressors, such as UV radiation, air pollutants, and toxic substances, combined with intrinsic factors such as chronological aging and hereditary effects, cause a detrimental condition known as oxidative stress. The increases in intracellular reactive oxygen species (ROS) levels can cause damaging changes in cellular macromolecules—proteins, lipids, carbohydrates, and nucleic acids—while also disrupting cellular mechanisms and signaling pathways. This affects skin health, leading to impaired function and premature aging processes, which manifest as wrinkles, loss of firmness, and various dermatological concerns [1].

Fibroblasts, essential constituents of the dermis, enhance the skin’s integrity by synthesizing and sustaining extracellular matrix (ECM) molecules. Oxidative stress impairs fibroblast cells by triggering inflammatory responses and inducing cellular injury [2]. The elevation of intracellular ROS levels triggers the JNK/ERK mitogen-activated protein kinase (MAPK) pathway, which in turn activates the downstream transcription factor c-Jun [3]. This factor dimerizes with c-Fos to augment AP-1 activity. Additionally, ROS can promote the phosphorylation of Akt and MAPK, consequently stimulating NF-κB inflammatory pathways [4]. The activation of AP-1 and NF-κB leads to an increase in the expression of pro-inflammatory cytokines and pro-apoptotic proteins [5]. These alterations result in cellular damage and the degradation of the dermal layer’s ECM, expediting skin aging [6]. Thus, targeting oxidative stress in anti-skin aging should be prioritized.

Mitigating excessive ROS is essential for safeguarding skin cells and decelerating the aging process. Several studies have examined the role of antioxidants in plants, such as herbs and fruits, in reducing oxidative stress and enhancing skin health. Antioxidant-rich plants encompass many phytochemical substances, including phenolic acids, flavonoids, triterpenoids, carotenoids, and vitamins, and could attenuate skin damage by neutralizing internal ROS. Consequently, solutions using natural plants to protect against fibroblast oxidative stress show considerable potential.

*Gymnema inodorum* (GI), or Pak Chaing Da, is indigenous to Northern Thailand, where its leaves are frequently used in local cuisine and relied upon in traditional herbal medicine to lower blood glucose levels [7]. GI extracts (GIEs) were reported to have promising antihyperglycemic activities by reducing glucose intake and α-glucosidase activity [8], which come across with the application of this plant for anti-diabetic purposes. Gymnemic acid, a triterpenoid found in its leaves, delays glycemic intake by improving glucose absorption through insulin mimicry [9], inhibition of α-glucosidase [10], and suppression of sodium-glucose cotransporter type 1 (SGLT1) [11]. GIE reduces obesity by enhancing lipase activity and inhibiting the differentiation of adipocytes [7,12]. This effect also improves glucose intolerance and dyslipidemia in rodents on a high-fat diet [13]. GIE enhances anti-inflammatory effects via reducing the TNF-α/NF-κB signaling pathway. [14,15]. Furthermore, GIE has exhibited antimalarial properties and protective effects on various organ systems in *Plasmodium berghei*-infected mice [16,17,18,19,20,21]. In terms of antioxidants, there is a strong relationship between phytochemicals (phenolics, flavonoids, quercetin, and kaempferol) found in GIE and antioxidant activity (Oxygen radical absorbance capacity (ORAC), Ferric-reducing antioxidant power (FRAP), 1,1-Diphenyl-2-picrylhydrazyl (DPPH), and 2,2′-azino-bis(3-ethylbenzothiazoline-6-sulfonic acid (ABST) assay) [22,23]. The high potential in antioxidant and inflammation of GIE was reported to be involved in H_2_O_2_-induced endothelial cell death and hypoxia-induced cardiomyocyte injury [24]. The phytochemicals found in GIE were reported in several studies, including phenolic acids, flavonoids, vitamins, terpenoids and saponins, and other compounds [12,22,23,25,26].

The beneficial effect and the presence of various phytochemical antioxidants in GIE suggest that it is effective in preventing cells from oxidative stress. However, the investigation of GIE on oxidative stress in dermal skin fibroblasts and the specific mechanism involved remain unexamined. Therefore, this study aims to investigate the antioxidant properties of six different ethanol leaf extracts of GI and compare their preventive effects against oxidative stress in human dermal fibroblast cells with a commercial native Gymnema extract (NGE). The phytochemical compositions of these extracts were also analyzed, including total phenolics, total flavonoids, and total triterpenes, as well as their antioxidant capacity, specific antioxidant scavenging activity, and anti-glycation properties. Subsequently, selection of extracts for further investigation into the prevention of fibroblast oxidative damage were based on their antioxidant potential. The highest antioxidant potential extracts, including GIE0, GIE80, GIE95, and NGE, were chosen to be examined for their preventive effects and underlying mechanisms through inflammatory cell signaling cascades (MAPK/AP-1/NF-κB and AKT/NF-κB) on H_2_O_2_-induced fibroblast cell oxidative stress. These extracts could help mitigate oxidative stress-induced cell injury, offering insights into their potential protective role at the cellular level, which could also support skin protection by preserving the health and function of dermal fibroblasts.

## 2. Materials and Methods

### 2.1. Chemicals

The chemicals used in this study were analytical grade or higher (≥95%); NaNO_2_, AlCl_3_, NaCO_3_, 2,4,6-tripyridyl-S-triazine (TPTZ), FeCl_3_, (±)-6-Hydroxy-2,5,7,8-tetramethylchromane-2-carboxylic acid (Trolox), FeSO_4_, 2,2,-azobis(2-methylpropionamidine) dihydrochloride (AAPH), Fluorescein, Thiobarbituric acid (TBA), 2-deoxy-D-ribose, Trichloroacetic acid (TCA), Nitroblue tetrazolium (NBT), Phenazine methosulfate (PMS), Nicotinamide adenine dinucleotide (NADH), 5,5′-dithiobis(2-nitrobenzoic acid) (DTNB), sodium borohydride (NaBH_4_), aminoguanidine (AG), NaN_3_, methylglyoxal (MGO), Hydrogen peroxide, oleanolic acid (OAE), 2′,7′-Dichlorofluorescein diacetate (DCFH-DA), and the other chemicals were obtained from Sigma-Aldrich (St. Louis, MO, USA). Type 1 ultrapure water was obtained using the Smart2Pure™ Water Purification System (Thermo Fisher Scientific, Waltham, MA, USA), with a resistivity of 18.2 MΩ·cm at 25 °C and a Total Organic Carbon (TOC) level of ≤10 ppb. Bio-Rad protein assay and polyacrylamide were obtained from Bio-Rad Laboratories Ltd. Cell cultures, including Dulbecco’s Modified Eagle Medium (DMEM), fetal bovine serum (FBS), trypsin–EDTA solution, RIPA buffer, (3-(4, 5-dimethylthiazolyl-2)-2, 5-diphenyltetrazolium bromide) (MTT), and cell culture supplies (dishes, plates, and others), were obtained from Gibco^®^ (Thermo Fisher Scientific, Waltham, MA, USA). All the antibodies for the Western blot experiments were obtained from Cell Signaling Technology (Danvers, MA, USA).

### 2.2. Gymnema Inodorum Extract (GIE) and Native Gymnema Extract (NGE) Preparation

The GI leaves were obtained from the Chiang-Da farm at the Agricultural Technology Research Institute, Lumpang Province, Thailand (geocoordinates: 18.352710° N, 99.600657° E). Young and medium-matured leaves of GI were desiccated in a vacuum oven at 40 °C. Two grams of dried GI leaves were soaked in 60 mL of either water (GIE0) or ethanol–water mixtures containing 20% (GIE20), 40% (GIE40), 60% (GIE60), 80% (GIE80), or 95% (GIE95) ethanol (*v*/*v*), maintaining a solid-to-solvent ratio of 1:30. Extraction was performed using ultrasound-assisted extraction at a frequency of 37 kHz and 100% power at 50 °C for 60 min. Following extraction, the resulting solution was filtered and concentrated using a rotary evaporator. The concentrated solution was lyophilized to provide a powder with an approximate grain size of 250 µm and thereafter stored at 4 °C. A native Gymnema extract (NGE), an ethanolic extract of *Gymnema sylvestre*, was obtained from the USP store (CAS No. 1302258, San Diego, CA, USA). Each extract was dissolved in 50% DMSO in water (*v*/*v*) to create a stock solution at a concentration of 100 mg/mL of dried extract powder before being diluted and undergoing testing in each method. Different ethanol leaf extracts of GI were compared to a commercial NGE, chosen for its distinct species classification within the same genus, well-documented antioxidant properties, empirical support, and commercial availability.

### 2.3. Measurement of Phytochemical Constituents

#### 2.3.1. Determination of Antioxidant Compounds by High-Performance Liquid Chromatography (HPLC)

Chromatographic analysis of the sample solution was performed using a Waters chromatograph (Milford, MA, USA) equipped with an Alliance (Hillside, IL, USA) 2695 separation module and a 2998 photodiode array detector. Data were analyzed using Empower Pro 2 Software (Waters, MA, USA). In brief, 20 µL of the standard mixture solution (containing quercetin, kaempferol, and GiA 1) or GI extracts was injected into an Atlantis^®^ RP-C18 column (250 × 4.6 mm, 5 µm), equipped with a SecurityGuard™ C18 cartridge (4 mm × 2.0 mm, Phenomenex, Inc., Bangkok, Thailand), and the column was maintained at 25 °C. Two mobile phases were used for chemical separation: mobile phase A (0.5% (*v*/*v*) phosphoric acid and 10 mM potassium dihydrogen phosphate in ultrapure water) and mobile phase B (acetonitrile). Gradient elution was performed over 20 min, linearly transitioning from 75% to 25% mobile phase A. The composition was then increased from 25% to 40% A over 5 min, held at 40% A for another 5 min, before returning to 75% A over the final 10 min. The flow rate was set at 1.6 mL/min. Detection wavelengths were set at 264 nm for kaempferol and quercetin, and 210 nm for kaempferol, quercetin, gallic acid, coumaric acid, ferulic acid, and Gymnemic acid 1 (GiA 1). Linear ranges, regression equations, and recoveries for quercetin, kaempferol, and GiA 1 are presented in Table S1.

#### 2.3.2. Determination of Total Flavonoids

The total flavonoid content (TFC) was determined using the aluminum colorimetric technique, as described by Okolie et al. [27]. The GIE and NGE (final volume of 5 mL) were mixed with 0.3 mL of a 5% (*w*/*v*) NaNO_2_ in distilled water and incubated for 5 min in the dark at room temperature (25 °C) with intermittent shaking. Subsequently, 0.3 mL of 10% (*w*/*v*) AlCl_3_ in distilled water was added, and after 1 min, the solution was combined with 2 mL of 1M NaOH. The final volume was calibrated to 10 mL utilizing 2.4 mL of type 1 ultrapure water. The solution was thoroughly mixed, and the absorbance was measured at 510 nm using a UV–visible spectrophotometer. Quercetin served as the reference for the calibration curve, and the TFC of the extract was quantified as micromoles of quercetin per gram extract powder.

#### 2.3.3. Determination of Total Phenolic

The total phenolic content (TPC) was evaluated using the Folin–Ciocalteu colorimetric method as described by Mamelona et al. [28]. One milliliter of samples was combined with five milliliters of Folin–Ciocalteu reagent and four milliliters of 7.5% (*w*/*v*) sodium carbonate. Following a 30 min incubation at 40 °C, the absorbance was measured at 765 nm. Gallic acid was used to create the calibration curve, and the TPC of the extract was quantified as micromoles of gallic acid per gram extract.

#### 2.3.4. Determination of Total Triterpenoid 

A total triterpenoid content (TTC) in the extracts was determined by the colorimetric technique as outlined in [29,30]. Briefly, 100 µL of each methanol sample was combined with 150 µL of a 5% (*w*/*v*) vanillin–glacial acetic acid solution and 500 µL of 70% perchloric acid solution. The reaction mixture was incubated for 45 min at 60 °C and then cooled to room temperature. After adding 2.25 mL of glacial acetic acid, the absorbance was recorded at 548 nm. A calibration curve was performed with oleanolic acid (OAE) as the standard reference. The TTC of the extract was quantified as micromoles of oleanolic acid per gram extract. 

### 2.4. Determination of Antioxidant Potential

#### 2.4.1. Determination of Ferric-Reducing Antioxidant Power (FRAP) Assay 

The antioxidant potential of the sample was determined using the FRAP assay as described by Benzie and Strain [31]. This test relies on the reducing power of antioxidant compounds capable of converting Fe^3+^-TPTZ (iron[III]-2,4,6-tripyridyl-S-triazine) to Fe^2+^-TPTZ, a vibrant blue complex, therefore enhancing absorbance at 593 nm. Briefly, the FRAP reagent was prepared by mixing 10 mM TPTZ in 40 mM HCl, 20 mM FeCl_3_ in 40 mM HCl, and 300 mM sodium acetate buffer (pH 3.6) at 1:1:10 (*v*/*v*/*v*) in a 96-well plate. Ten microliters of samples and two hundred microliters of FRAP reagent were thoroughly mixed into each well, and then the mixture was incubated at room temperature. After 5 min, a Synergy H1 multi-mode reader (Biotek, Winooski, VM, USA) was employed to measure an absorbance at 593 nm. FeSO_4_ and Trolox were used as standards at concentrations from 0 to 1 mM for the calibration curve preparation. The FRAP value of each sample was determined using the linear regression equation y = ax + b of FeSO_4_ (*y*-axis) and sample concentrations (*x*-axis). The antioxidant capacity of the extracts was evidenced as micromoles of FeSO_4_ per gram extract. 

#### 2.4.2. Determination of Oxygen Radical Absorbance Capacity (ORAC) Assay

The ORAC assay, as described by Cao et al. [32], is commonly utilized to evaluate the extract’s antioxidant capacity, and its resulting values are used for comparing the antioxidant potential of different foods and other substances. This method is based on the fluorescence quenching of fluorescein after it reacts with peroxyl free radicals, which are generated from a compound produced by AAPH. Performing the assay, 25 µL of various concentrations of standard and sample in 75 mM potassium phosphate buffer pH 7.4 was mixed with 150 µL of 7.5 nM fluorescein in 75 mM potassium phosphate buffer pH 7.4 and incubated at 37 °C for 5 min. Then, the reaction was initiated by adding 25 µL of 165 mM AAPH in 75 mM potassium phosphate buffer pH 7.4, and the fluorescence intensity was measured at 485 nm excitation and 528 nm emission wavelengths every minute for a duration of 90 min. The areas under the curve (AUCs) of plotting graphs between percent inhibition and time (minutes) were measured. The net AUC was calculated following the equation

Net AUC = AUC_sample_ − AUC_blank_

The dose–response data plot between the sample’s net AUC values (*x*-axis) and the net AUC values of Trolox (*y*-axis) was utilized to calculate the ORAC values of each sample. The resulting ORAC values were expressed as Trolox equivalent (TEq) in micromoles per gram extract.

### 2.5. Determination of Specific Antioxidant Scavenging Activity 

#### 2.5.1. Hydroxyl Radical Scavenging Activity Assay

The hydroxyl radical scavenging activity of extracts was determined by the colorimetric method as described by Halliwell et al. [33]. This assay is based on the production of OH^•^ via the Fenton reaction, which oxidizes 2-deoxy-D-ribose and produces malondialdehyde (MDA), a recognized indicator of oxidative stress. Subsequently, MDA interacts with thiobarbituric acid (TBA), forming the MDA-TBA adduct. Measuring the pink chromogen allows for the evaluation of the extract’s capacity to scavenge OH^•^. The assay was performed by combining a solution containing a final concentration of 2 mM EDTA, 0.1 mM FeCl_3_, 1.12 mM 2-deoxy-D-ribose, 0.2 mM H_2_O_2_, and 0.2 mM sodium L-ascorbic acid with a variety of samples and standard concentrations in 0.1 M potassium phosphate buffer pH 7.4. The solution was then incubated at 50 °C for 20 min. The reaction was terminated by the addition of 1.12% (*w*/*v*) trichloroacetic acid (TCA), followed by the formation of MDA-TBA adducts by the combination of the solution with 0.4% TBA (*w*/*v*) in 0.5% NaOH and incubation at 100 °C for 15 min. Following the cooling of the mixture, the absorbance at 530 nm was quantified utilizing a Synergy H1 multi-mode reader (Biotek, Winooski, VM, USA). The % inhibition is determined by comparing the reaction with the extract to the response without the extract (Blank). The equation for percent inhibition was determined using the following formula:

Percent inhibition = [(Absorbance of control − Absorbance of sample)/Absorbance of control] × 100%

The IC_50_ value is established by plotting percent inhibition against the logarithm of sample concentration and applying a sigmoidal dose–response curve to the data. The log(IC_50_) value derived from the curve was subsequently transformed into the relevant concentration (IC_50_) for documentation.

#### 2.5.2. Superoxide Anion Scavenging Activity Assay

The capacity of extracts to scavenge O_2_^•−^ was assessed via the decrease in NBT, using the methodology outlined by Nishikimi et al. [34]. Superoxide anions are generated when PMS and NADH interact, resulting in the reduction of NBT to a blue formazan product that enhances absorbance at 560 nm. For the test, 50 µL of diluted samples (0–8 µg/mL) and a standard Trolox (0–8 µM) in 20 mM potassium phosphate buffer at pH 7.4 were introduced into each well of the 96-well plate. Subsequently, 90 µL of 172 µM NBT in distilled water and 30 µL of 600 µM NADH in 20 mM potassium phosphate buffer at pH 7.4 were added and gently agitated at ambient temperature for 5 min on the shaker. The reaction began by combining 30 µL of 60 µM PMS in each well and incubating for 5 min with moderate agitation. Following incubation, the formazan production was quantified at an absorbance wavelength of 560 nm using a Synergy H1 multi-mode reader (Biotek, Winooski, VM, USA) and the percentage of inhibition was determined using the equation

Percent inhibition = [(Absorbance of control − Absorbance of sample)/Absorbance of control] × 100%

The IC_50_ value is established by plotting percent inhibition against the logarithm of sample concentration and applying a sigmoidal dose-response curve to the data. The log(IC_50_) value derived from the curve was subsequently transformed into the relevant concentration (IC_50_) for documents.

#### 2.5.3. Hypochlorous Acid Scavenging Activity Assay

The HOCl scavenging technique outlined by Ching et al. [35] involves the oxidation of 5-thio-2-nitrobenzoic acid (TNB), which turns yellow, into DTNB colorless, upon reaction with HOCl, resulting in a reduction of absorbance at 412 nm. Briefly, TNB solution was prepared by mixing 1.2 mM DTNB in 50 mM potassium phosphate buffer pH 6.6 with 120 mM NaBH_4_ in type 1 ultrapure water at a ratio of 5:1 (*v*/*v*). The mixture was subsequently vortexed using Vortex-Genie 2 (Scientific Industries, Bohemia, NY, USA) to remove the gas produced during the preparation process. To achieve a concentration of 40 µM TNB, the absorbance at 412 nm was measured, and then the concentration was adjusted as required using the molar absorption coefficient of TNB at 13,600 M^−1^cm^−1^. HOCl was prepared by diluting a commercial NaOCl solution (4.00–4.99% available chlorine, Sigma-Aldrich (St. Louis, MO, USA)) with distilled water and adjusting the pH to 6.2 with 0.6 M H_2_SO_4_. The HOCl concentration was calibrated to 40 µM according to the molar absorption coefficient, established at 100 M^−1^cm^−1^ at an absorbance wavelength of 235 nm. Initially, the samples and standards were combined with a 40 µM TNB solution, and the absorbance was recorded at 412 nm before and 5 min after the addition of 40 µM HOCl using a Synergy H1 multi-mode reader (Biotek, Winooski, VM, USA). The percentage of inhibition in each sample was determined using the following equation:

Percent inhibition = [Absorbance before adding HOCl − Absorbance after adding HOCl)/Absorbance before adding HOCl] × 100%

The IC_50_ value is established by plotting percent inhibition against the logarithm of sample concentration and applying a sigmoidal dose-response curve to the data. The log(IC_50_) value derived from the curve was subsequently transformed into the relevant concentration (IC_50_) for documents.

#### 2.5.4. Hydrogen Peroxide Scavenging Activity Assay

This method evaluates the extract’s ability to mitigate the detrimental effects of H_2_O_2_, as described by Ruch et al. [36]. Briefly, 0.1 mL of extracts and standards with concentrations ranging from 0.5 to 8 mg/mL were added to microcentrifuge tubes. The volume of each extract was increased to 0.8 mL by adding 50 mM phosphate buffer with a pH of 7.4. The reaction began by combining 0.2 mL of 40 mM H_2_O_2_, then followed by vortexing. Following a reaction period of 10 min at room temperature without agitation, the absorbance was measured at wavelength 230 nm using a Synergy H1 multi-mode reader (Biotek, Winooski, VM, USA). The percentage of inhibition was determined according to the equation:

Percent inhibition = [(Absorbance of control − Absorbance of sample)/Absorbance of control] × 100%

The IC_50_ value is established by plotting percent inhibition against the logarithm of sample concentration and applying a sigmoidal dose–response curve to the data. The log(IC_50_) value derived from the curve was subsequently transformed into the relevant concentration (IC_50_) for documents.

### 2.6. Bovine Serum Albumin (BSA)–Methylglyoxal (MGO) Glycation Inhibitory Activity Assay

This experiment assesses the anti-glycation activity of extracts using a BSA-MGO reaction model as described by Wu and Yen [37]. Inhibition of advanced glycation end products (AGEs) production was assessed through the glycation of BSA by MGO, evaluating the extract’s potential. Performing the method, extracts (0–1000 µg/mL) and standard aminoguanidine (AG) (0–10 mM) dissolved in 100 mM potassium phosphate buffer pH 7.4 containing 0.02% NaN_3_ (*w*/*v*) were mixed with 20 mg/mL of BSA Fraction V (Sigma-Aldrich, St. Louis, MO, USA) and 60 mM of MGO, respectively, in a microcentrifuge tube at a ratio of 1:1:1. Then, solutions were vortexed at medium speed for 30 s and subsequently incubated at 37 °C for 7 days in the dark. After that, the fluorescence intensity of each sample was measured at excitation (340 nm) and emission (420 nm) wavelengths using a Synergy H1 multi-mode reader (Biotek, Winooski, VM, USA), and percent inhibition was calculated using the following equation:

Percent inhibition = [(Fluorescence intensity of control − Fluorescence intensity of sample)/Fluorescence intensity of control] × 100%

IC_50_ values were then calculated from the plot between the log of sample concentration and percent inhibition.

### 2.7. Cell Culture and Treatment

Human primary dermal fibroblast, HDFa (PCS-201-012), obtained from ATCC (Manassas, VA, USA), was cultured in Dulbecco’s Modified Eagle Medium (DMEM) supplemented with 10% fetal bovine serum (FBS) within the Forma™ Series II Water-Jacketed CO_2_ Incubator (Thermo Fisher Scientific, Waltham, MA, USA) maintained at 37 °C under a humidified atmosphere of 5% (*v*/*v*) CO_2_. The culture medium was renewed every three days, and the cells were subcultured for experiments once they reached 80–90% confluence. In the treatment protocol, cells were grown in culture plates for 24 h and then pretreated by adding the fresh media containing GIE0, GIE85, GIE95, and NGE concentrations ranging from 1 to 1000 µg/mL and incubated for 24 h. After being removed and washed with phosphate-buffered saline (PBS), oxidative stress was generated by adding fresh media containing 1–4 mM H_2_O_2_ for a duration of 1 to 4 h. The treated cells were subsequently produced according to the protocol of each experiment.

### 2.8. Cell Viability Assay

The preventive effect of GIE and NGE was evaluated by a 3-(4,5-dimethylthiazol-2-yl)-2,5-diphenyl tetrazolium bromide (MTT) cell viability assay. We seeded HDFa cells at a density of 1 × 10^4^ cells per well in 200 μL of complete DMEM per well in a 96-well cell culture plate and incubated them for 18–24 h at 37 °C in a humidified incubator with 5% (*v*/*v*) CO_2_. Subsequently, new media with diverse concentrations of GIE and NGE (1, 10, 50, 100, 500, and 1000 µg/mL) were subjected to a 24 h pretreatment. After washing with 1× PBS, the cells were treated with or without 1 mM H_2_O_2_, diluted in DMEM, for 2 h. Cells were then washed with 1× PBS and incubated with DMEM containing 0.25 mg/mL MTT for 3 h. After incubation, the media were removed and then the produced formazan crystals were solubilized in 100% DMSO by adding 100 µL in each well. The plate was shaken for 10 min, and their intensity was quantified at an absorbance wavelength of 550 nm utilizing a SpectraMax^®^ M2e microplate reader (Molecular Devices, San Jose, CA, USA). The toxicity of GIE and NGE (24 h) and H_2_O_2_ (1–4 h) on fibroblast cells was followed by the same methodology. The findings are shown as a percentage of cell viability relative to the vehicle-treated group.

### 2.9. Determination of Intracellular ROS

The preventive effects of GIE and NGE on H_2_O_2_-induced fibroblast cell oxidative stress were determined using an intracellular ROS probe, 2′,7′-Dichlorofluorescein diacetate (DCFH-DA). Cells were seeded in a 35 mm cell culture dish at a density of 2 × 10^5^ cells/dish in 2 mL of complete DMEM. The cells were cultured for 18–24 h at 37 °C in a humidified incubator with 5%. Medium was carefully removed by aspiration. Subsequently, new media with diverse concentrations of GIE and NGE (2 mL of 50 µg/mL) was subjected to a 24 h pretreatment. After washing with 1× PBS, the cells were treated with or without 2 mL of 1 mM H_2_O_2_, diluted in DMEM, for 2 h. Treated cells were washed with 2 mL of 1× PBS and incubated with 2 mL of medium containing 25 μg/mL DCFH-DA for 30 min at 37 °C in a humidified incubator with 5% (*v*/*v*) CO_2_. After incubation, the media were removed and washed with 1× PBS. The fibroblast cell oxidative stress was observed under the Olympus FluoView FV10i confocal laser scanning microscope (Olympus Corporation, Shinjuku, Tokyo, Japan) using excitation and emission wavelengths of approximately 490 nm and 525 nm, respectively. ImageJ software, version 1.54 was used to quantify the percentage of mean fluorescence intensity of intracellular ROS. The proportion of mean fluorescence intensity was used to compare the intracellular ROS levels using the vehicle-treated group (cells treated with the same final concentration of DMSO as the treated group, but without extract) as a reference. 

### 2.10. Western Blot Assay

HDFa cells were seeded in 60 mm cell culture dishes at 2 × 10^5^ cells/well density in 3 mL of complete DMEM. The cells were cultured for 18–24 h at 37 °C in a humidified incubator with 5% (*v*/*v*) CO_2_. Subsequently, new media with diverse concentrations of GIE and NGE (3 mL of 10 and 50 µg/mL) was subjected to a 24 h pretreatment. After washing with 1× PBS, the cells were treated with or without 3 mL of 1 mM H_2_O_2_, diluted in DMEM, for 0.5–2 h. For post-treatment, proteins were extracted using RIPA lysis buffer and nuclear extraction buffer. In summary, treated cells were rinsed twice with 1× ice-cold PBS (maintaining on ice at all times), subsequently removed, and then resuspended in 200 µL of lysis buffer. The supernatant of each sample was transferred to a microcentrifuge tube and incubated for 30 min. Subsequently, lysates were centrifuged at 12,000× *g* for 30 min. The protein lysate concentrations were quantified with the Bio-Rad protein assay, which involved the addition of 10 µL of sample and standard BSA to 200 µL of Bradford solution, followed by absorbance measurement at 595 nm using a Synergy H1 multi-mode reader (Biotek, Winooski, VM, USA). The protein lysate in each sample was adjusted to ensure equal loading (20 µg per lane) using sample buffer and 5× loading dye (Bio-Rad Laboratories Ltd., Hercules, CA, USA) prior to separation via SDS-PAGE. The separating gels (10%) were prepared by combining deionized water, a 30% (*w*/*v*) acrylamide–bisacrylamide (29:1) solution (Bio-Rad, Hercules, CA, USA), 1.5 M Tris-HCl (pH 8.8), 10% SDS, 10% ammonium persulfate, and TEMED. Stacking gels were prepared in a similar manner, utilizing 0.5 M Tris-HCl (pH 6.8) as the buffer solution. Gel electrophoresis was performed using a running buffer composed of 25 mM Tris, 192 mM glycine, and 0.1% SDS at pH 8.3. A voltage of 100 V was applied for 1.5 h with a Mini-PROTEAN^®^ system (Bio-Rad, Hercules, CA, USA). Proteins separated in polyacrylamide gel were transferred to Amersham Hybond P 0.45 PVDF blotting membranes (GE Healthcare, Chicago, IL, USA) with a mini-PROTEAN Tetra system and PowerPac™ HC power supply (Bio-Rad Laboratories Ltd., Hercules, CA, USA). The nonspecific protein-binding sites on blotted membranes were obstructed using a 5% (*w*/*v*) blocking solution of BSA or nonfat dried milk in tris-buffered saline with 0.1% (*v*/*v*) Tween^®^ 20 detergent (TBST) for 1 h. Subsequently, the blocked membranes were treated overnight with primary antibodies diluted to 1:1000 in blocking buffer, including NF-κB (#4764), β-actin (#3700), Phospho-AKT (#4051), Phospho-SAPK/JNK (#4668), Phospho-p44/42 MAPK (Erk1/2) (#4370), and Phosphor-c-Jun (#9164). Following three washes (10 min each) with 10 mL of 1× TBST, the swollen membranes were incubated for 1 h with 1:3000 anti-rabbit (#7076) or anti-mouse (#7074) IgG conjugated with horseradish peroxidase (HRP). Following a wash with 1× TBST, the protein bands on the membranes were probed using Amersham ECL Select Western Blot Reagent and detected with a GE Healthcare Amersham Imager 600 Series Gel Documentation System (GE Healthcare, Chicago, IL, USA). The bands were quantified and analyzed using ImageJ software. 

### 2.11. Statistical Analysis

Statistical analysis of this study was conducted using GraphPad Prism software (version 10) The results are presented as the mean ± standard error of the mean (SEM), derived from a minimum of three distinct measurements. Linear and non-linear (curve fit) regressions were employed to analyze the dose–response data curves in the trials. Principal component analysis (PCA) and a heat map correlation matrix utilizing the Pearson correlation coefficient (r) were employed to identify the significant relationships between elements contributing to the overall variability of the examined research procedures data. For PCA, the analysis included data from Table 1, specifically the measurements of phytochemical constituents, total antioxidant capacity, ROS scavenging activity, and anti-glycation activity, all of which were analyzed using GraphPad Prism. One-way ANOVA was used to investigate differences across different groups based on a single factor, whereas two-way ANOVA was applied to evaluate the interaction and main effects of two independent variables. Comparisons between the two groups were performed using the *t*-test. A *p*-value below 0.05 was considered statistically significant.

## 3. Results

### 3.1. Phytochemical Constituents, Antioxidant Potential, Specific ROS Scavenging Activity, and Anti-Glycation of GIE and NGE

The HPLC-based phytochemical screening profile of each GIE is shown in Figure S1 and Tables S1 and S2, where quercetin, kaempferol, and GiA 1 were detected with retention times of 9.8, 13.0, and 14.4 min, respectively, while no peaks corresponding to gallic acid, coumaric acid, or ferulic acid were observed. The integration of analyte signals was performed using a retention time window of ±0.05 min around each peak maximum. Table S2 presents the concentrations of quercetin, kaempferol, and GiA 1 in crude extracts prepared using varying quantities of ethanol. The 0% ethanol extract lacked quercetin and kaempferol but included GiA 1 at a concentration of 0.70 ± 0.14 mg per 100 mg of crude extract. As the ethanol concentration increased, the levels of quercetin and kaempferol also rose, with the 95% ethanol extract showing the highest concentration of 37.0 ± 0.98 µg and 29.60 ± 0.99 µg per 100 mg of crude extract, respectively. Similarly, the concentration of GiA 1 increased with rising ethanol concentrations, peaking at 2.40 ± 0.10 mg per 100 mg of crude extract in the 80% ethanol extract.

Table 1 presents a comprehensive summary of the bioactive components, antioxidant activities, reactive oxygen species scavenging capacities, and anti-glycation properties of both GIE and NGE. The phytochemical analysis revealed significant variation in total phenolic content (TPC) among the extracts. The TPC values showed no significant differences among GIE group; nevertheless, they were elevated compared to the NGE. GIE exhibited a higher TPC compared to NGE, likely due to the commercial extract’s focus on triterpenes such as Gymnemic acids, which may not be ideal for the extraction of phenolic compounds, as indicated by the USP Certificate. The total flavonoid content (TFC) in GIE increased with the percentage of ethanolic solvent, with GIE95 exhibiting the highest TFC, followed by GIE80 and GIE60, exceeding the values of GIE40, GIE20, NGE, and GIE0, respectively. Additionally, the correlation between the ethanolic solvent percentage of GIE and total triterpenoid content (TTC) revealed a descending order, with GIE95 showing the highest value, followed by GIE80, GIE60, GIE40, GIE20, and GIE0, respectively. NGE exhibited a higher TTC than all GIE. 

The antioxidant capacity of extracts was evaluated using FRAP and ORAC methods. The result revealed FRAP values (micromoles of FeSO_4_ per gram extract) among the extracts; NGE demonstrated the highest antioxidant potential, followed by GIE80, GIE0, GIE95, GIE40, GIE60, and GIE20, arranged in descending order based on their significance. The ORAC values (micromoles of Trolox per gram extract) of the extracts were arranged in descending order of significance, with NGE exhibiting the highest value, followed by GIE80, and then GIE0 and GIE40, which were higher than GIE60, GIE95, and GIE20, respectively. 

The specific scavenging activities of OH^•^, O_2_^•−^, HOCl, and H_2_O_2_ by the extracts demonstrated their ability to neutralize free radicals. The extracts’ ability to scavenge OH^•^ radicals was arranged in descending order of IC_50_ values (µg/mL extract), with GIE0, GIE80, and NGE exhibiting the highest activity, followed by GIE40 and GIE95, which were higher than GIE20 and GIE60, respectively. Another radical, O_2_^•−^ scavenges by extracts ranked from NGE, GIE0, GIE40, and GIE20, followed by GIE60, GIE95, and GIE80, respectively. The scavenging activity of extracts on HOCl revealed that GIE95 exhibited the highest potential, followed by GIE80, GIE0, and NGE, which were greater than GIE60, GIE40, and GIE20, respectively, arranged in descending order based on their significance. Furthermore, the extract’s properties to scavenge H_2_O_2_ were ranked, with NGE, GIE0, and GIE80 exhibiting higher potential compared to GIE40, GIE95, and GIE60, with GIE20 showing the lowest activity, respectively. 

The anti-glycation potential of extracts was evaluated by measuring their ability to protect BSA against glycation induced by MGO, resulting in decreased formation of AGEs. Dose–response relationships were observed in the percent inhibition of AGE formation with increasing concentrations of the standard anti-AGE inhibitor, AG. The extract’s inhibitory potential against protein glycation was further evaluated by calculating their IC_50_ values, showing that GIE95 and GIE80 exhibited the highest anti-glycation activity compared to GIE60 and GIE40, followed by NGE, then GIE0, and GIE20, respectively, arranged in descending order based on their significance.

### 3.2. Principal Component Analysis (PCA) and Heat Map Correlation Matrix 

**Figure 1:**
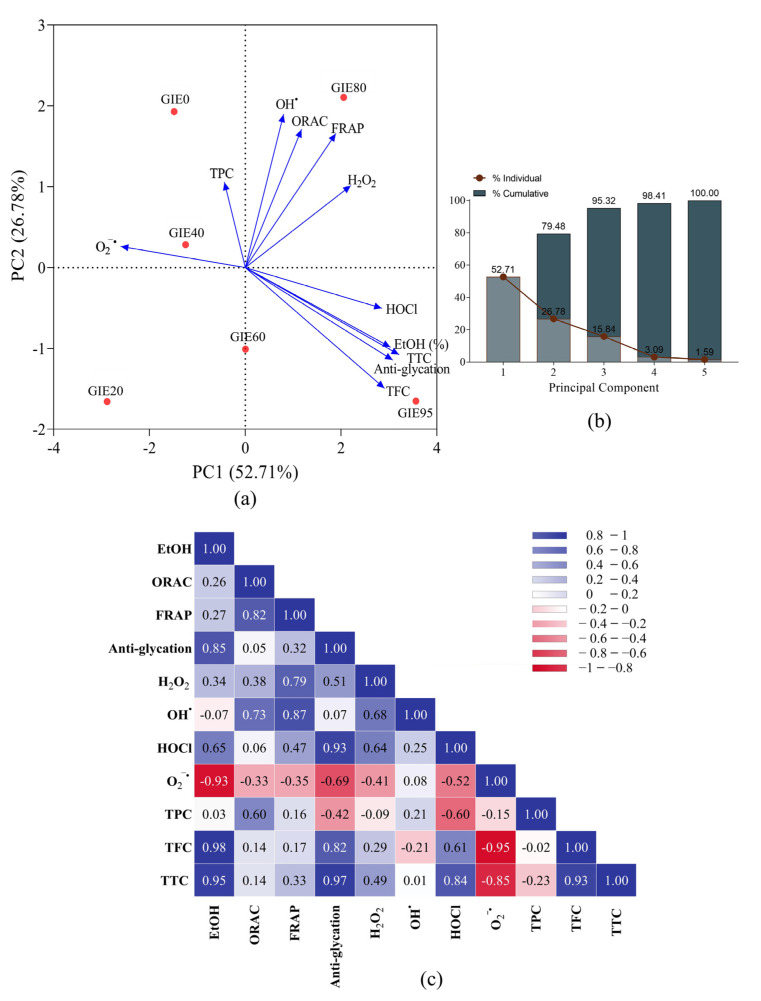
The study of parametric analysis for GIE and determination methods: (**a**) Principal component analysis (PCA) of studied parameters. (**b**) Proportion of variance in PCA, both individually and cumulatively (**c**). Heat map of Pearson’s correlation coefficient matrix. The analytical data served as the standard equivalent of 1 g extract, and Pearson’s correlation coefficient was utilized with a significance threshold of *p* < 0.05.

PCA was conducted to assess the variation in the percentage of GI ethanolic extract (ranging from 0% to 95%), phytochemical contents, and their potential for antioxidant and anti-glycation activities. The results indicate that PC1 and PC2 individually account for 52.71% and 26.78% of the total variance (cumulatively 79.48%), respectively, for GIE (Figure 1b). As represented in Figure 1a and Table S3, PC1 is positively associated with TTC (3.206); TFC (2.902); percent ethanolic extract of GI (3.026); HOCl (2.846), H_2_O_2_ (2.200), and OH^•^ (0.798) scavenging activities; FRAP (1.888); ORAC (1.173); and anti-glycation (3.074), while PC2 is positively correlated with ORAC (1.708), FRAP (1.656), OH^•^ (1.895), H_2_O_2_ (1.012) scavenging activity, and TPC (1.055). The reduced space of the two first principal components is utilized to visualize all GIEs. Notably, GIE95 (3.562) and GIE80 (2.053) were positively characterized by PC1. The high PC1 scores for GIE95 and GIE80 are attributed to their high TTC and TFC contents, suggesting that these extracts are potential candidates for antioxidant, anti-glycation, and scavenging activities against HOCl and H_2_O_2_. Moreover, GIE0 (1.929) and GIE80 (2.105) are positively characterized by PC2, indicating their elevated TPC and significant antioxidant capacities as measured by ORAC, FRAP, and scavenging activities for OH^•^ and H_2_O_2_. Nevertheless, GIE40, GIE60, and GIE20 have negative values for both PC1 and PC2 in the GIE group. Therefore, GIE0, GIE80, and GIE95, along with NGE, were used to evaluate their preventive effects on dermal fibroblast cell oxidative stress.

The heat map correlation matrix in Figure 1c provides valuable insights into the interplay among different factors, including the percentage of ethanolic extract, phytochemical constituents, antioxidant capacity, and anti-glycation activity in GIE. TTC, TFC, anti-glycation activity, and HOCl scavenging ability demonstrated positive associations with the increased utilization of the percent ethanolic solvent for GI extraction. Extracts rich in TTC and TFC content exhibited a promising capacity to mitigate protein glycation and counteract the production of harmful HOCl. Turning our attention to antioxidant assays, our findings reveal compelling associations. The positive correlations between FRAP and ORAC (r = 0.82), H_2_O_2_ (r = 0.79), and OH^•^ (r = 0.87) scavenging activities highlight their significant contributions to the overall antioxidant capacity, as evaluated through GIE. 

### 3.3. Toxicity of GIE and NGE on HDFa Cells

**Figure 2:**
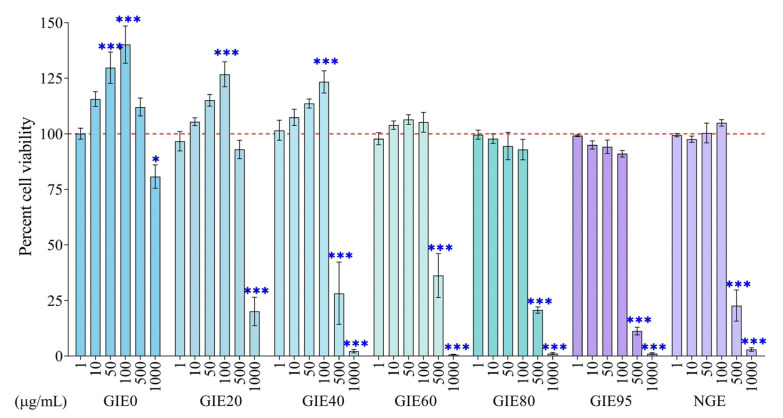
Toxicity of GIE, NGE, and H_2_O_2_ treatment on fibroblast cell. Cytotoxic effect of various concentrations of GIE and NGE on the cell viability of fibroblast cells. Data are shown as mean ± SEM of n ≥ 3. * *p* < 0.05, and *** *p* < 0.001 when compared to the vehicle-treated group.

The toxicity of extracts to fibroblast cells was determined using a MTT cell viability assay. Treatment with GIE (0–95) and NGE at 1–100 µg/mL dosages for 24 h had no harmful effects on HDFa cells until exceeding 500 µg/mL (Figure 2). Compared to the vehicle-treated group (100%), GIE0 (130 ± 7% and 140 ± 8%) and GIE20 (115 ± 3% and 126 ± 6%) exhibited an increase in cell viability at concentrations of 50 and 100 µg/mL. We utilized GIE0, GIE80, GIE95, and NGE in our investigation of the preventative effect of GIE on fibroblast oxidative stress since they demonstrated strong antioxidant capacity using PCA and antioxidant activity methods. Referring to their toxic effect, concentrations ranging from 1 to 100 µg/mL were chosen for this study. 

The oxidative stress induction in HDFa cells was performed using treatment with H_2_O_2_, in which its toxicity to the fibroblast cell was displayed in survival curves (Figure S3). The results showed that there was no cytoxicity effect of H_2_O_2_ at concentrations of 0.25 and 0.5 mM. However, treatment with an increasing concentration of H_2_O_2_ at 1, 2, and 4 mM for 1–4 h showed a significant decrease in cell viability. Exposure of fibroblasts with 1 mM of H_2_O_2_ for 1, 2, 3, and 4 h showed significant decreases in cell viability to 92.91 ± 0.60%, 65.76 ± 0.54%, 58.17 ± 1.10%, and 33.72 ± 1.13%, respectively, when compared to the control group (100%). Acute effect was observed when the cells were exposed to H_2_O_2_ at concentrations of 2 and 4 mM for 1–4 h, of which over 50% showed a reduction in survival cells. Based on these findings, a dose of 1 mM of H_2_O_2_ with an incubation time of 2 h was selected for studying the preventive effect of GIE and NGE on fibroblast oxidative stress. This choice was made because a reduction in cell viability of about 30–40% was deemed appropriate for assessing the efficacy of the extracts in mitigating oxidative stress-induced damage.

### 3.4. Preventive Effect of GIE and NGE on Intracellular ROS Production and Cell Viability in H_2_O_2_-Induced Fibroblast Injury and Oxidative Stress

**Figure 3:**
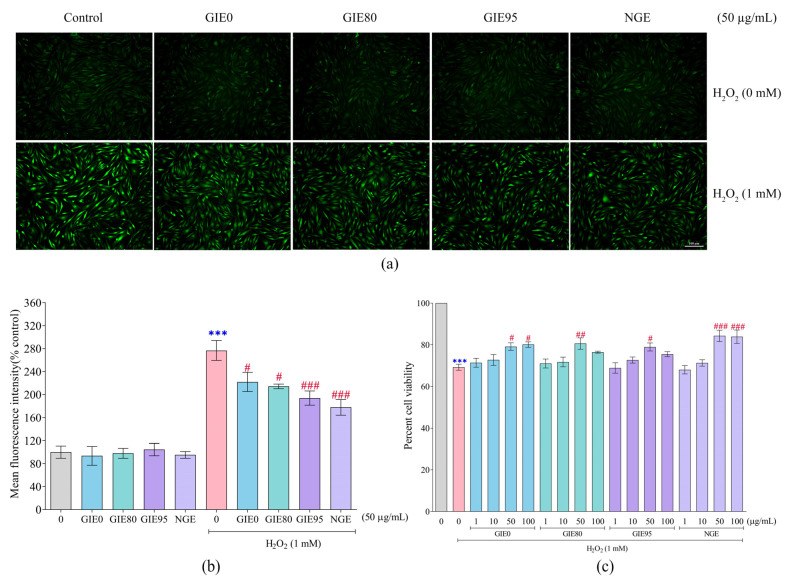
Preventive effect of GIE and NGE on H_2_O_2_-induced fibroblast oxidative stress and cell viability. (**a**) Representative picture of intracellular ROS, identified by DCFH-DA green fluorescence. (**b**) Effects of GIE and NGE on intracellular ROS production induced by H_2_O_2_. (**c**) Effects of GIE and NGE on H_2_O_2_-decreased cell viability. Data are presented as mean ± SEM of n ≥ 3. *** *p* < 0.001 when compared to the vehicle-treated group; # *p* < 0.05, ## *p* < 0.01, and ### *p* < 0.001 when compared to the H_2_O_2_-challenged group.

The effect of GIE and NGE on fibroblast oxidative stress was performed using a DCFH-DA probe. Figure 3a represents the observation of fluorescence in cells, where its intensity indicates intracellular ROS levels and demonstrates oxidative stress in the sample. The fibroblast cells treated with GIE0 (93.68 ± 10.69%), GIE80 (98.08 ± 16.44%), GIE95 (104.60 ± 10.92%), and NGE (95.31 ± 5.86%) have no significant difference in intracellular ROS levels when compared to the vehicle-treated group (100%). Conversely, HDFa cells exposed to 1 mM H_2_O_2_ demonstrate a rise in mean fluorescence intensity to 276.76 ± 17.31%. Pretreatment with GIE0, GIE80, and GIE95 markedly reduced intensity to 221.99 ± 16.57%, 214.31 ± 3.99%, and 193.86 ± 12.46%, respectively. Likewise, pretreatment with NGE led to a reduction in mean fluorescence intensity to 177.81 ± 13.52% in comparison to the H_2_O_2_-challenged group (Figure 3b).

The preventive effect of GIE and NGE on H_2_O_2_-challenged fibroblasts was examined. Pretreatment with GIE0 at concentrations of 50 and 100 µg/mL significantly increased percent cell viability to 79.16 ± 1.77% and 80.10 ± 1.29%, respectively, when compared to the vehicle-treated group (69.23 ± 1.84%). In the same way, pretreatment with 50 µg/mL of GIE80 and GIE95 in fibroblast cells exposed to H_2_O_2_ also remarkably relieved cells to 80.59 ± 2.71% and 78.96 ± 1.20%, respectively. Moreover, cell viability was notably increased to 84.28 ± 2.72% and 83.89 ± 3.24% when pretreated with NGE at concentrations of 50 and 100 µg/mL, respectively (Figure 3c).

### 3.5. Effect on Cell Signaling Pathways in H_2_O_2_-Induced Fibroblast Oxidative Stress

We measured the fold changes in several proteins in fibroblasts exposed to 1 mM H_2_O_2_ from 0 to 120 min based on band intensity on Western blot (Figure S4a). Exposure to H_2_O_2_ enhanced the cytosolic transduction proteins, including AKT, ERK, and JNK, as early as 30 min before decreasing at 120 min. The phosphorylation of AKT significantly increased to 13.18 ± 3.26-fold after the H_2_O_2_ challenge at 30 min (Figure S4b). MAPK signaling through ERK and JNK reached its maximum level at 30 min, with relative phosphorylation increases of 2.13 ± 0.07- and 14.92 ± 1.35-fold (*p* < 0.001), respectively (Figures S4c and S4d). Regarding downstream phosphorylation by AKT and MAPK, the inflammatory mediator NF-κB significantly increased after challenging H_2_O_2_ at 15, 30, 60, and 120 min to 1.36 ± 0.08-, 1.26 ± 0.04-, 1.22 ± 0.08-, and 1.29 ± 0.08-fold, respectively, when compared to the vehicle-treated group (Figure S4e). Moreover, the phosphorylation of c-Jun, which operates downstream from JNK, was substantially enhanced to 2.04 ± 0.28- and 2.00 ± 0.32-fold (*p* < 0.05) at 60 and 120 min, respectively (Figure S4f). As a result, the highest peak expression time point was chosen to further analyze the effects of GIE on each signaling molecule in H_2_O_2_-induced fibroblast oxidative stress during the time-course investigation.

### 3.6. Effect of GIE and NGE on Cell Signaling Pathways in H_2_O_2_-Induced Fibroblast Oxidative Stress

**Figure 4:**
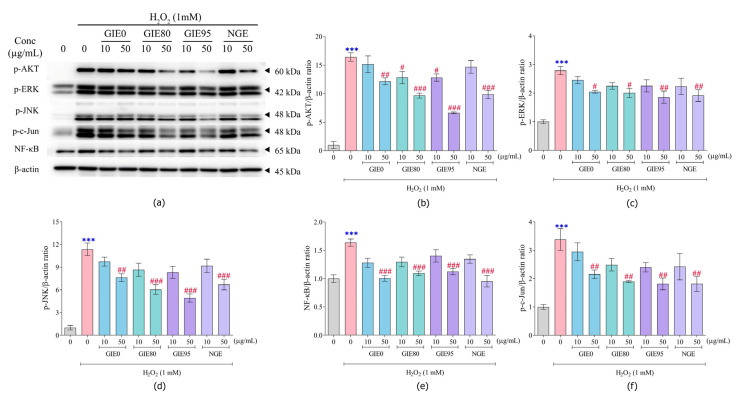
Effect of GIE and NGE pretreatment on p-AKT, p-ERK, p-JNK, NF-κB, and p-c-Jun signaling pathways in H_2_O_2_-induced fibroblast cell signaling changes in fibroblast cells exposed to H_2_O_2_. (**a**) Representative Western blot bands of each signaling protein. (**b**) p-AKT/β-actin. (**c**) p-ERK/β-actin. (**d**) p-JNK/β-actin. (**e**) NF-κB/β-actin. (**f**) p-c-Jun/β-actin. Data are presented as mean ± SEM of n ≥ 3. *** *p* < 0.001 when compared to the vehicle-treated group; # *p* < 0.05, ## *p* < 0.01, and ### *p* < 0.001 when compared to the H_2_O_2_-challenged group.

The protein bands of p-AKT, p-ERK, p-JNK, NF-κB, p-c-JUN, and housekeeping protein β-actin are shown in Figure 4a. Pretreatment with 50 µg/mL of GIE0, GIE80, GIE95, and NGE significantly decreased the p-AKT/β-actin ratio to 12.19 ± 0.60, 9.68 ± 0.43, 6.66 ± 0.16, and 9.86 ± 0.72, respectively, when compared to the H_2_O_2_-challenged group (16.45 ± 0.74) (Figure 4a,b). Similarly, the cytosolic transduction MAPK (ERK and JNK) was significantly decreased by pretreatment of GIE0 (2.04 ± 0.05 and 7.63 ± 0.49), GIE80 (2.01 ± 0.15 and 6.05 ± 0.64), GIE95 (1.86 ± 0.21 and 4.92 ± 0.54), and NGE (1.91 ± 0.20 and 6.70 ± 0.70) at concentrations of 50 µg/mL when compared to the exposed group (2.79 ± 0.14 and 11.36 ± 0.83) (Figure 4a,c,d). Further, the pretreatment of these extracts significantly reduced NF-κB signaling to 1.00 ± 0.05, 1.09 ± 0.04, 1.12 ± 0.06, 1.12 ± 0.06, and 0.96 ± 0.10, respectively, when compared to the H_2_O_2_-induced group (1.74 ± 0.07) (Figure 4a,e). Similarly, pretreatment of 50 µg/mL of GIE0, GIE80, GIE95, and NGE significantly decreased p-c-JUN/β-actin to 2.15 ± 0.15, 1.90 ± 0.03, 1.81 ± 0.21, and 1.82 ± 0.27, respectively, when compared to the H_2_O_2_-challenged group (3.39 ± 0.38) (Figure 4a,f).

## 4. Discussion

Repeated exposure to environmental and intrinsic factors accelerates skin aging via fibroblast oxidative stress. This ROS-driven process results in functional impairment, cellular damage, inflammation, and injury [38]. Antioxidants play an important role in mitigating ROS-induced oxidative damage, highlighting the importance of protective agents in skin health. Natural GIE and NGE, rich in bioactive phytochemicals, exhibit potent antioxidant and anti-inflammatory properties, offering a promising strategy to counteract skin oxidative stress and injury. By mitigating oxidative stress caused by free radicals, which are unstable compounds that can damage cells and accelerate the aging process, antioxidants are crucial for safeguarding our skin from the damage of aging [39].

This study identified the significant antioxidant potential of GIE, attributed to its rich content of phytochemical antioxidants, including flavonoids, phenolic acids, and triterpenoids, aligned with those documented by Nuchuchua O et al. [22], Muhammad H et al. [26], and Jeytawan N et al. [23]. Furthermore, we observed a direct correlation between the ethanol concentration during extraction and the yield of total triterpenoids and flavonoids in GIE, except phenolic compounds showed no such correlation, which is consistent with findings from leaf extracts of *B. davidii* and rice husk extracts [40,41]. GIE had a greater TPC than NGE, probably because the commercial extract targets triterpenes such Gymnemic acids, which may not be optimal for extracting phenolic compounds, as reported by the USP Certificate. Phenolic compounds exhibit varying polarity, and the ethanol–water ratio, the nature of the plant matrix, and the extraction conditions influence their extraction efficiency [42]. These phytochemical antioxidants assist in mitigating oxidative stress and its complications in disease development [43]. The presence of these natural antioxidant compounds in GIE renders them beneficial in preventing oxidative damage and the progression of its related diseases. These phytochemicals present in the GIE and their biological functions have been reported, including phenolic acids (coumarinic acid, feruloylquinic acid, caffeoylquinic acids, gallic acid, caffeic acid, vanillic acid, ferulic acid, chlorogenic acid), flavonoids (kaempferol, quercetin, isoorientin, catechin, myricetin), vitamins (tocopherol, ascorbyl stearate), terpenoids and saponins (ginsenoside, gymnemasaponin, triterpenoid, Gymnemic acid, saikosaponin, momordin, GiA 7), and other compounds (squalene, adenosine, phytol, stigmasterol, stephanoside, malic acid, propanoic acid, theobromine) [12,22,23,25,26]. Phytochemical screening of GIE by HPLC in this study identified antioxidant phytochemicals, including kaempferol and quercetin, consistent with Nuchuchua O et al. [44]. The identification of GiA 1 reveals its potential anti-adipogenic effect [12] and modulate glucose absorption [45], suggesting a protective effect against insulin resistance and diabetes.

The presence of large levels of biologically active compounds also indicated significant antioxidant action. This study employed FRAP and ORAC to assess antioxidant capacities. Among the GIE groups, GIE80 had the greatest FRAP values at 18.13 mmol TE/100 g extract, surpassing blackberry’s value of 3.99 mmol TE/100 g extract by a ratio of 4.54, whereas NGE reported a value of 29.68 ± 1.94 mmol TE/100 g extract, exceeding GIE by a fold of 1.63. The high FRAP values of plants indicate beneficial effects on skin health and the aging process, suggesting the potential use of plants like GIE in preventing these processes [46]. Therefore, GIE and NGE could serve as natural antioxidants and anti-aging agents for the skin. Moreover, the FRAP values of our GI-dried leaf extracts subjected to ultrasound-assisted extraction (UAE) (181.33 μmol TE/g) surpassed those documented by Nuchuchua et al. (59.06 to 119.36 μmol TE/g) [22] and were significantly greater than the fresh leaf extracts reported by Jeytawan (3.40 μmol TE/g) [23]. Furthermore, the UAE (45.38 mg TE/g) in this investigation was shown to be superior to ethanol maceration (16.00 mg TE/g) and ethanol reflux extraction (24.00 mg TE/g), although inferior to aqueous decoction (75.10 mg TE/g) and microwave-assisted aqueous extraction (52.00 mg TE/g) [26]. The evidence demonstrated the efficacy of the extraction procedure utilizing UAE in this investigation [47].

ORAC is commonly used to evaluate antioxidant capacities; a higher ORAC score indicates a more potent antioxidant, and the FDA recommends consuming foods with an ORAC level of 3000–5000 daily for optimal health [48]. The antioxidant trend observed in FRAP is also reflected in this test, where the ORAC value of GIE80 (2091.53 μmol TE/g) is the highest among our GIE. Our GIE surpassed that of 75% ethanolic GIE from Prachin Buri (455.96 ± 12.65), Fang and Mae Tang Chiang Mai (558.27 and 1843.45 μmol TE/g), and Chiang Rai (840.61 μmol TE/g) [22]. Moreover, the ORAC value of NGE (3557.89 μmol TE/g) is about 1.7 times higher than GIE, while the lemon polyphenol (5400 μmol TE/g) [49] and orange (6074 μmol TE/g) are about 2.6 and 2.9 times, respectively [50]. Lemon and orange extracts have been incorporated into cosmetics, including skin conditioners and balms, to maintain the health of the skin [51,52]. The efficacy of GIE and NGE suggests that they have skin-protective properties and could be used as components in dermal protection products.

A substantial increase in intracellular ROS is the hallmark of skin cell responses to extrinsic factors, including UV irradiation and pollution. The specific ROS formed, including H_2_O_2_, OH^•^, HOCl, and O_2_^•−^, promote cellular oxidative stress, resulting in various signal transduction cascades linked with cellular changes and injury [53]. GIE and NGE were discovered to effectively scavenge these ROS, although their efficacy against each oxidant varied. This may be due to the complex interactions of the extracted compounds. Scavenging these oxidants with extracts is essential for protecting skin cells from oxidative damage. For instance, O_2_^•−^ is predominantly generated in the mitochondria during cellular respiration and by NADPH oxidase. It can directly damage biomolecules and interact with other molecules to produce H_2_O_2_, contributing to inflammation and destroying lipids, proteins, and DNA [54]. OH^•^ is mostly generated from H_2_O_2_ by the Fenton reaction. This radical is highly reactive and can rapidly harm cell membranes and biomolecules, which may result in mutations, cellular apoptosis, or carcinogenesis [55,56]. The HOCl is synthesized by myeloperoxidase and H_2_O_2_, inducing chlorinative stress, by the chlorination of cellular components, impairing their function and causing chronic inflammation and tissue damage [57]. These ROS are converted to others through intracellular mechanisms [58]. Owing to their high scavenging capacity to inhibit all types of ROS, GIE and NGE can thoroughly regulate the entire intracellular ROS pathway, making them effective regulators of oxidative stress.

Glycolytic overload causes dicarbonyl stress, the accumulation of reactive dicarbonyl compound methylglyoxal (MGO), which contributes to metabolic syndromes and chronic diseases, cardiovascular disease, and cancer [59]. MGO is a major precursor of AGEs, which degrade proteins and other macromolecules, promoting oxidative stress and worsening metabolic dysfunction [60]. High efficacy in inhibiting this irreversible process would optimally safeguard cells, with GIE80 and GIE95 being the most effective for anti-glycation in this study. The considerable potential of GIE in anti-glycation research highlights its importance as a viable area for future studies in this field of research.

Pearson’s correlation coefficient matrix was employed to investigate the correlation between the efficacy of GIE in each method. The increased utilization of the percent ethanolic solvent for GI extraction was positively correlated with TTC, TFC, anti-glycation activity, and HOCl scavenging ability. Extracts that were abundant in triterpenoid and flavonoid compounds demonstrated a promising ability to reduce protein glycation and prevent the production of deleterious HOCl. Our findings indicate persuasive associations when we focus on antioxidant assays. The substantial contributions of FRAP to the overall antioxidant capacity, as assessed by GIE, are underscored by the positive correlations between ORAC, H_2_O_2_, and OH^•^ scavenging activities. PCA was applied to identify and choose the most effective antioxidants among GIE. Our result showed that PC1 has a positive correlation with TTC; the percentage of ethanolic extract of GIE, anti-glycation, TFC, HOCl and H_2_O_2_ scavenging activities, FRAP, ORAC, and OH^•^, whereas PC2 is favorably associated with ORAC, FRAP, OH^•^, H_2_O_2_ scavenging activity, and TPC. This implies that the data point shifts toward PC1 indicated high antioxidant activity, same with Nuchuchua O et al. [22], whereas the shift toward PC2 reflected a combination of both antioxidant and anti-glycation properties. PCA analysis, which includes NGE as presented in Figure S2, indicates that NGE exhibits high antioxidant potential. However, data from NGE are excluded from the primary objective of comparing different GIEs. Incorporating NGE into the PCA may lead to interpretations that deviate from the intended comparative framework of GIE variants, due to its unique species origin and extraction methodology. This study focuses on the antioxidant potential to prevent dermal fibroblast oxidative stress. Therefore, only high antioxidant potential extracts were selected to assess their preventative impact on fibroblast oxidative damage.

The cytotoxicity of GIE and NGE was varied at concentrations of 1–1000 µg/mL. We found that some concentrations (50–100 μg/mL) of GIE0, GIE20, and GIE40 significantly increased in cell viability percentage (Figure 2). The increase in cell viability could be attributed to the provision of essential nutrients and bioactive compounds by plant extracts, which can influence the signaling pathways that regulate cell proliferation and differentiation [61]. Although enhancing cell growth is an intriguing aspect of wound healing and other research fields, this study focuses exclusively on PCA linked to the highest antioxidant potential (GIE0, GIE80, GIE95, and NGE). In examining these extracts, the toxic dosages of extracts were excluded, and the selected concentrations were 1, 10, 50, and 100 μg/mL. H_2_O_2_ is commonly used in research to create oxidative stress in cell culture models. The reason for using H_2_O_2_ instead of the UV exposure model is because UV not only induces oxidative stress, but also causes DNA damage and provokes inflammation, limiting the isolation of the extract’s antioxidant activities. Therefore, induction by H_2_O_2_ serves as a more direct oxidative stress model for investigating antioxidant effects. We optimized the HDFa oxidative damage model by varying H_2_O_2_ concentration (0–4 mM) and incubation time (1–4 h). To evaluate the prevention of extracts, 1 mM of H_2_O_2_ induction for 2 h was selected because of cell death of about 30–40% control following our previous study [62]. Oxidative stress arises in compromised cells due to an increase in ROS. Our research corroborates the findings previously published by Buranasudja V. et al., indicating that the H_2_O_2_-induced fibroblast cell model exhibited elevated intracellular ROS levels [63]. Excessive oxidative stress impairs biomolecules and promotes fibroblast inflammation and injury, whereas phyto-antioxidants serve as essential agents to mitigate H_2_O_2_-induced damage by attenuating oxidative stress [64]. For instance, Lee S et al. have reported that the anti-aging effect of inotodiol on H_2_O_2_-induced human dermal fibroblast oxidative stress is achieved by reducing ROS accumulation, which in turn results in a decrease in the sensing of inflammatory pathways [65]. Likewise to the research conducted by Hahn H, rosmaric acid inhibits inflammatory response and cellular senescence through NF-κB [66]. This study demonstrated that GIE0, GIE80, GIE95, and NGE reduced intracellular ROS production and enhanced cell survival in dermal fibroblasts under oxidative stress. The cytoprotective action of GIE could originate from its antioxidant core components, as shown in Table 1. Previous studies reported the protective effect of GIE on cardiomyocytes [24] and endothelial cell injury [22] by decreasing intracellular ROS levels and enhancing cell survival. Thus, our research suggests that GIE and NGE have the potential to prevent fibroblast injury and aging under their antioxidant properties.

The intracellular mechanism triggered by ROS promotes inflammation and damage in dermal fibroblasts [67]. Accumulation of ROS invokes skin cell injury via MAPK/NF-κB/AP-1 signaling pathways [68]. In dermal fibroblasts, the redox-sensitive members of the MAPK family, including ERK, JNK, and p38, are activated by ROS, leading to the subsequent activation of c-Jun/AP-1 and NF-κB. These transcription factors stimulate the production of inflammatory cytokines and matrix metalloproteinases (MMPs), leading to cellular injury and collagen degradation during skin aging [69]. In this study, we observed that H_2_O_2_ induces injury in HDFa by rapidly increasing the phosphorylation of MAPK (ERK and JNK), leading to the downstream activation of p-c-Jun and the NF-κB transcription factor. Pretreatment with either GIE0, GIE80, GIE95, or NGE (50 µg/mL) diminishes the phosphorylation of ERK and JNK, along with the activation of critical inflammatory transcription factors, p-c-Jun and NF-κB. Similarly, research conducted by Dunkhunthod B et al. indicated that GIE mitigates oxidative stress and the production of inflammatory mediators in RAW264.7 macrophages by downregulating the NF-κB signaling pathway and decreasing the release of pro-inflammatory cytokines [14]. In a similar vein, Surinkaew S et al. reported that GIE demonstrates anti-inflammation properties that protect against cardiomyocyte injury [24]. Consequently, GIE could help to mitigate fibroblast cell injury caused by oxidative stress by inhibiting the inflammatory signaling cascade, specifically the MAPK/AP-1/NF-κB axis. Furthermore, the activation of PI3K/AKT plays a significant role in protecting the fibroblast from damage and injury induced by ROS [70]. This study demonstrated the stimulation of AKT phosphorylation early point (30 min), which was diminished by GIE0, GIE80, GIE95, and NGE. Chronic activation of AKT is associated with the progression of cancer-associated fibroblasts [71]. Thus, the effect of GIE and NGE on the preservation of AKT phosphorylation levels in fibroblast oxidative stress might provide anti-cancer activity from the perturbation of the PI3K/AKT signal. Crosstalk is also observed in the activation of the PI3K/AKT and NF-κB signaling pathways, which promote the release of inflammatory cytokines [72]. Besides their anticancer properties, GIE and NGEs also mitigate fibroblast oxidative stress by regulating the PI3K/AKT/NF-κB pathway. The presence of antioxidants, including polyphenols, in GIE and NGE exhibits anti-aging properties in aged human dermal fibroblasts, primarily through these mechanisms, as reported in the recent publication [73].

**Figure 5:**
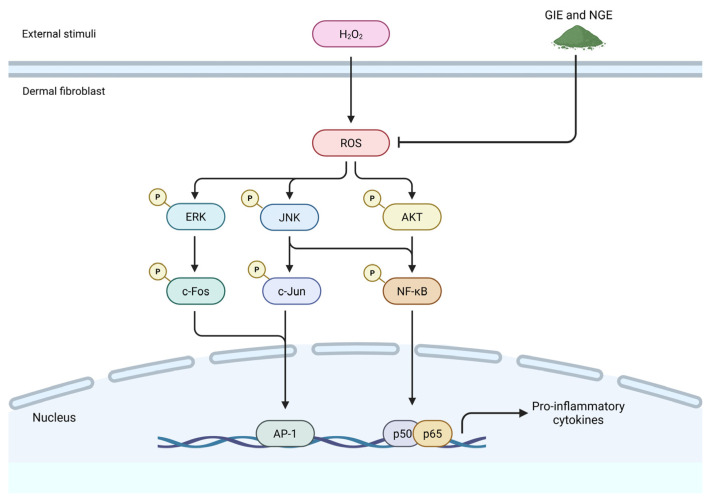
Graphical representation of the proposed mechanism of GIE and NGE preventing skin fibroblast oxidative stress induced by H_2_O_2_.

In summary, GIE0, GIE80, GIE95, and NGE showed the most antioxidant capacity and were chosen to examine the preventive effect on fibroblast cell oxidative stress, analyzed by PCA. The scavenging activities of GIE and NGE play a critical role in maintaining the balance of excessive ROS and ensuring redox homeostasis, which are the key mechanisms for preventing fibroblast inflammation and damage caused by oxidative stress. The redox chain of the MAPK/AP-1/NF-κB and PI3K/AKT/NF-κB signaling cascades is disrupted by a decrease in ROS accumulation, resulting in a decline in the production of inflammatory cytokines. This, in turn, reduces inflammation and injury in fibroblasts (Figure 5). GIE and NGE show potential advantages in mitigating fibroblast inflammation and injury in a model of oxidative stress induced by H_2_O_2_. We suggest the utilization of GIE0, GIE80, GIE95, and NGE for antioxidant purposes, whereas the application of GIE80 and GIE95 in terms of anti-glycation needs further research. More advanced in vivo and clinical research is required to clarify the efficacy of these dietary supplements in preventing fibroblast damage, skin aging, or cancer-associated fibroblasts.

This study’s limitations encompass the employment of in vitro models, which may not accurately reflect the behavior of the extracts in vivo, and the dependence on H_2_O_2_-induced oxidative stress, failing to consider additional external factors such as UV exposure or chronic inflammation. The findings cannot be directly applied to clinical practice due to the absence of long-term data and in vivo confirmation. In addition, as our study focused primarily on mechanistic pathways, we can only hypothesize about their potential roles in inflammation and injury. These aspects should be explored in greater detail in future studies. Subsequent research ought to employ in vivo animal models or clinical trials to demonstrate that GIE mitigates oxidative stress-related skin damage and ageing. Analyzing GIE under various stress conditions, assessing their long-term efficacy, and investigating their mechanisms through novel techniques is essential. GIE may be utilized in dietary supplements or cosmetics to mitigate oxidative damage, promote skin ageing, and enhance skin health. To establish the efficacy and safety of the extracts in medicinal and cosmetic applications, it is important to assess bioavailability, skin absorption, and toxicity, and to conduct human clinical trials.

## 5. Conclusions

GIE and NGE prevented H_2_O_2_-induced oxidative stress in fibroblast cells by scavenging intracellular ROS. The deactivation of MAPK/AP-1/NF-κB and PI3K/AKT/NF-κB signaling cascades leads to a reduction in cellular inflammation and injury. GIEs demonstrate potential for development as functional foods and nutraceuticals aimed at mitigating fibroblast injury, addressing the risks associated with skin oxidative damage and aging.

## Figures and Tables

**Table 1:** Extract phytochemical constituents, total antioxidant capacity, ROS scavenging activity, and anti-glycation activity.

Sample	Phytochemical Constituents			Total Antioxidant Capacity		ROS Scavenging Activity				Anti-Glycation
	TPC (µmol GAE/g)	TFC (µmol QE/g)	TTC (µmol OAE/g)	FRAP (µmol FeSO_4_/g)	ORAC (µmol Trolox/g)	OH^•^ (IC_50_ µg/mL)	O_2_^•−^ (IC_50_ µg/mL)	H_2_O_2_ (IC_50_ µg/mL)	HOCl (IC_50_ µg/mL)	MGO-Derived AGEs (IC_50_ µg/mL)
ASC	-	-	-	-	-	-	-	116.23 ± 1.9	111.6 ± 5.7	-
Trolox	-	-	-	-	-	37.8 ± 1.0	207.8 ± 9.1	204.79 ± 0.82	-	-
AG	-	-	-	-	-	-	-	-	-	23.57 ± 0.39
GIE0	317 ± 58 ^ab^	52.7 ± 3.5 ^a^	205 ± 38 ^a^	310. ± 4.1 ^d^	1417.1 ± 6.3 ^ab^	115.8 ± 2.6 ^e^	33.5 ± 1.6 ^b^	329 ± 21 ^cd^	66.23 ± 0.50 ^c^	883 ± 35 ^b^
GIE20	317 ± 24 ^b^	71.9 ± 3.5 ^ab^	213 ± 14 ^ab^	200.6 ± 2.5 ^a^	1091 ± 54 ^a^	174.4 ± 2.6 ^a^	54.5 ± 2.3 ^b^	568.1 ± 6.2 ^a^	146.0 ± 4.3 ^a^	1037 ± 52 ^a^
GIE40	334 ± 34 ^b^	78.6 ± 4.1 ^b^	307 ± 13 ^b^	241.4 ± 1.0 ^b^	1340 ± 210 ^ab^	143.2 ± 2.5 ^c^	38.5 ± 2.3 ^b^	475.29 ± 4.70 ^b^	118 ± 44 ^b^	608 ± 16 ^d^
GIE60	331 ± 17 ^b^	107.53 ± 0.28 ^c^	377 ± 15 ^bc^	240.5 ± 1.1 ^b^	1230 ± 140 ^a^	189.2 ± 8.9 ^a^	208 ± 18 ^a^	505.02 ± 0.51 ^b^	106.2 ± 3.9 ^b^	549 ± 15 ^d^
GIE80	338 ± 20 ^b^	109 ± 5.5 ^c^	455 ± 4.4 ^c^	340.5 ± 4.2 ^e^	2078 ± 43 ^b^	124.3 ± 1.9 ^de^	256 ± 19 ^a^	402 ± 16 ^c^	58.73 ± 0.89 ^c^	395 ± 13 ^e^
GIE95	304 ± 8.8 ^b^	127.6 ± 0.94 ^e^	643 ± 6.6 ^d^	277.7 ± 7.2 ^c^	1186 ± 34 ^a^	152.9 ± 2.7 ^abc^	255 ± 22 ^a^	493.4 ± 9.7 ^b^	37.5 ± 1.5 ^d^	327.3 ± 6.4 ^e^
NGE	204 ± 5.2 ^a^	60.9 ± 3.3 ^ab^	816 ± 12 ^e^	560.4 ± 11.5 ^f^	3560 ± 44 ^c^	129.6 ± 4.6 ^cde^	23.0 ± 7.1 ^b^	302.9 ± 4.6 ^d^	74.6 ± 4.7 ^c^	743 ± 12 ^c^

Note: Data are presented as the mean ± SEM. The ANOVA test was used to determine the statistical significance at *p* < 0.05. Superscripts indicate significant variations in means across samples in the same column, whereas identical letters indicate no significant difference.

## Data Availability

The data are contained within the article and Supplementary Materials.

## Supplementary Materials

The following supporting information can be downloaded at: https://www.mdpi.com/article/10.3390/antiox14091043/s1, Figure S1: HPLC analysis of phytochemical screening of different ethanolic extracts of GIE. Figure S2: Principal component analysis (PCA) of studied parameters of GIEs and NGE. Figure S3: Survival curve of fibroblasts challenged with H_2_O_2_ (0–4 mM) at time-course for 1–4 h. Figure S4: Time-course study of cell signaling changes in fibroblast cells exposed to H_2_O_2_. Figure S5: Original images of Western blot band intensities in Figure S4. Figure S6: Original images of Western blot band intensities in Figure 4. Table S1: Summary of linear calibration ranges, regression equations, coefficients of correlation (r^2^), detection limits (LOD), quantification limits (LOQ), and % recovery for quercetin, kaempferol, and GiA 1 analyzed by HPLC. Table S2: Quantification of quercetin, kaempferol, and GiA1 in crude extracts prepared with different ethanol concentrations by HPLC. Table S3: Factor loadings of each variable and principal component (PC) scores from principal component analysis (PCA).
