# Supplementary material for: Cytoprotective Effects of Gymnema inodorum Against Oxidative Stress-Induced Human Dermal Fibroblasts Injury: A Potential Candidate for Anti-Aging Applications
Source: Antioxidants (Basel). 2025 Aug 24;14(9):1043. doi: 10.3390/antiox14091043 (PMC12466490; doi:10.3390/antiox14091043)
Supplement: Supplementary file 1 [file antioxidants-14-01043-s001.zip › antioxidants-3760015-supplementary.pdf]

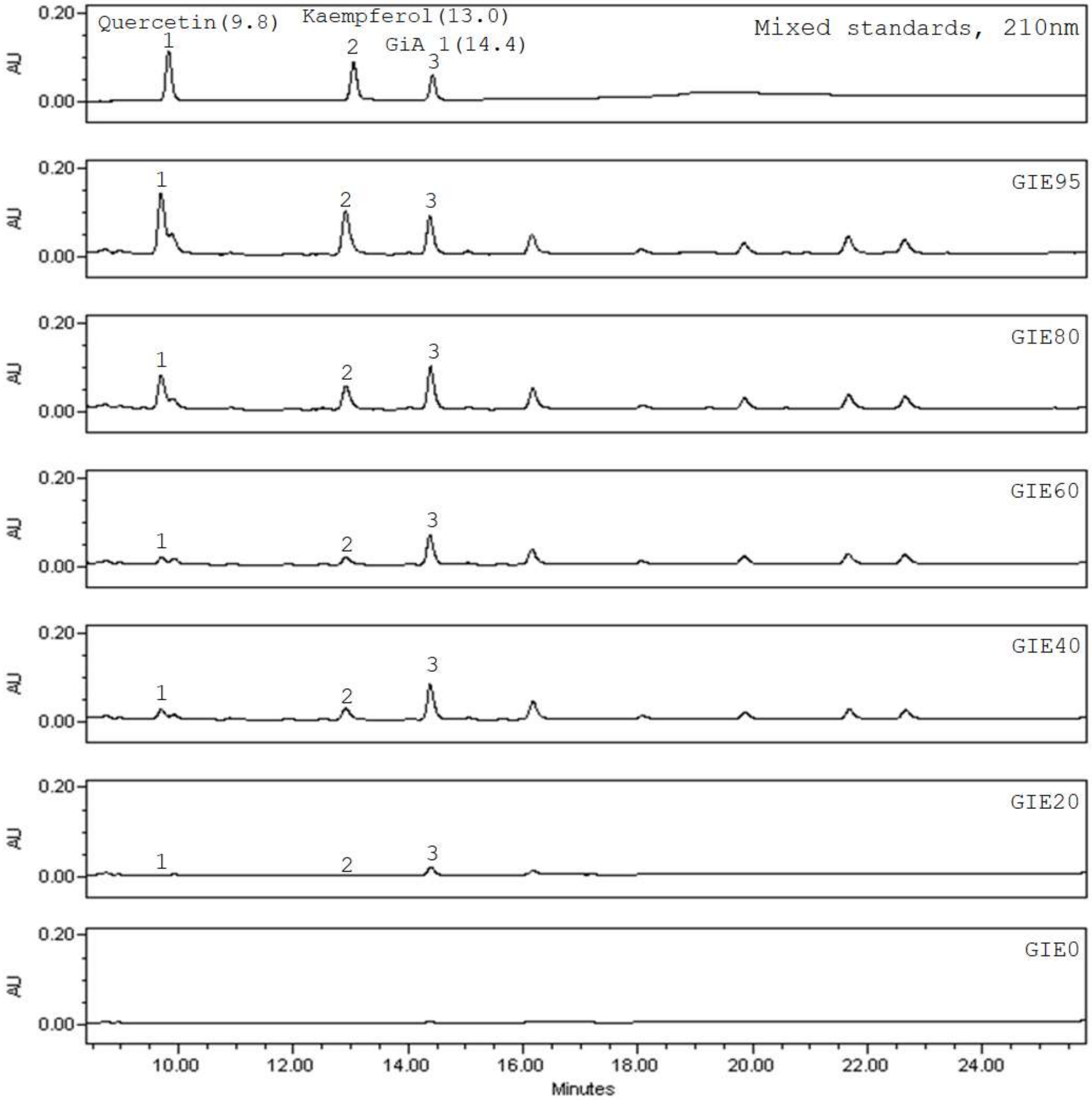

Figure S1 HPLC analysis of phytochemical screening of different ethanolic extracts of GIE.

Table S1 Summary of linear calibration ranges, regression equations, coefficients of correlation (r^2), detection limits (LOD), quantification limits (LOQ), and % recovery for quercetin, kaempferol, and GiA 1, analyzed by HPLC.

Compounds	Linear ranges (mg/mL)	Regression equations	Coefficient correlation, r^2	LOD (mg/mL)	LOQ (mg/mL)	%Recovery		
						Low level	Medium level	High level
Quercetin	0.001-0.060	y = 34,512,289.66x – 7,897.55	0.9998	0.0008	0.0023	95.34	104.55	94.61
Kaempferol	0.001-0.060	y = 44,484,074.73x + 2,841.60	0.9999	0.0002	0.0006	108.77	95.97	99.50
GiA 1	0.005-0.500	y = 3,629,581.09x – 2,520.28	0.9999	0.0021	0.0063	94.57	98.99	108.14

Table S2 Quantification of quercetin, kaempferol, and GiA 1 in crude extracts prepared with different ethanol concentrations by HPLC.

Sample Name	Concentration (per 100 mg crude extract)		
	**Quercetin, µg*** **(R_t=9.8 min)**	**Kaempferol, µg*** **(R_t=13.0 min)**	**GiA 1, mg** **(R_t=14.4 min)**
GIE0	-	-	0.696 ± 0.136
GIE20	2.700 ± 0.474	3.383 ± 0.669	1.815 ± 0.027
GIE40	6.384 ± 0.681	7.161 ± 0.378	2.139± 0.170
GIE60	20.028 ± 1.285	19.136 ± 1.283	2.108 ± 0.141
GIE80	26.439 ± 0.235	21.763 ± 0.218	2.408 ± 0.099
GIE95	36.944 ± 0.982	29.579 ± 0.989	2.260 ± 0.203

Table S3 Factor loadings of each variable and principal component (PC) scores from principal component analysis (PCA).

**Label**	**Group**	**PC1**	**PC2**	**PC3**
EtOH (%)	Loadings	3.025591	-0.98321	0.481527
ORAC	Loadings	1.172621	1.707634	0.512896
FRAP	Loadings	1.887931	1.656096	-0.49551
Anti-glycation	Loadings	3.074002	-1.14232	-0.68891
H_2O_2	Loadings	2.199859	1.012271	-1.23916
$OH^{\bullet}$	Loadings	0.798429	1.894888	-0.98755
HOCl	Loadings	2.845795	-0.50209	-1.66535
$O_2^{-\bullet}$	Loadings	-2.59662	0.261148	-1.46846
TPC	Loadings	-0.43606	1.055273	1.188702
TFC	Loadings	2.902352	-1.49165	0.497877
TTC	Loadings	3.20564	-1.07506	0.037107
GIE0	PC scores	-1.48587	1.929153	-1.85039
GIE20	PC scores	-2.88513	-1.65739	0.105759
GIE40	PC scores	-1.24686	0.283334	0.432488
GIE60	PC scores	0.002979	-1.00924	1.288596
GIE80	PC scores	2.053053	2.105431	1.32078
GIE95	PC scores	3.561823	-1.65129	-1.29724

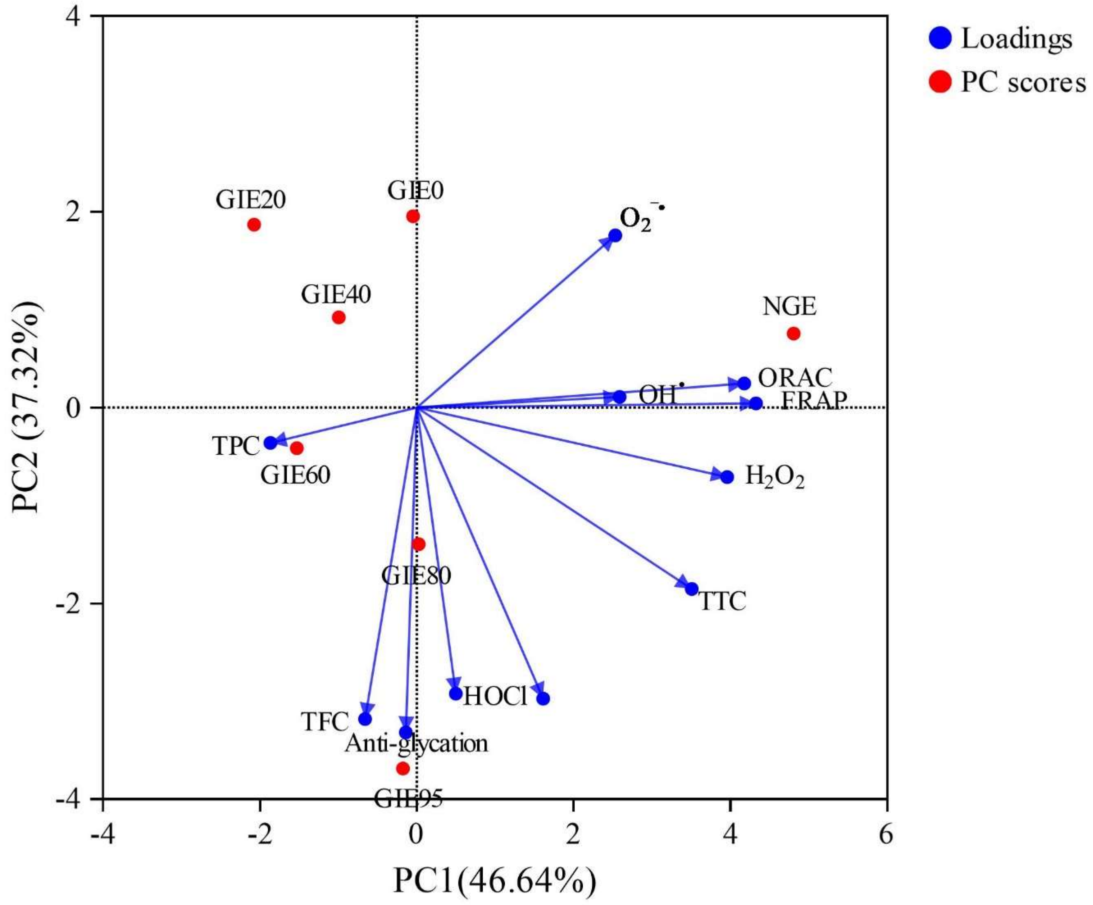

Figure S2 Principal component analysis (PCA) of studied parameters of GIEs and NGE.

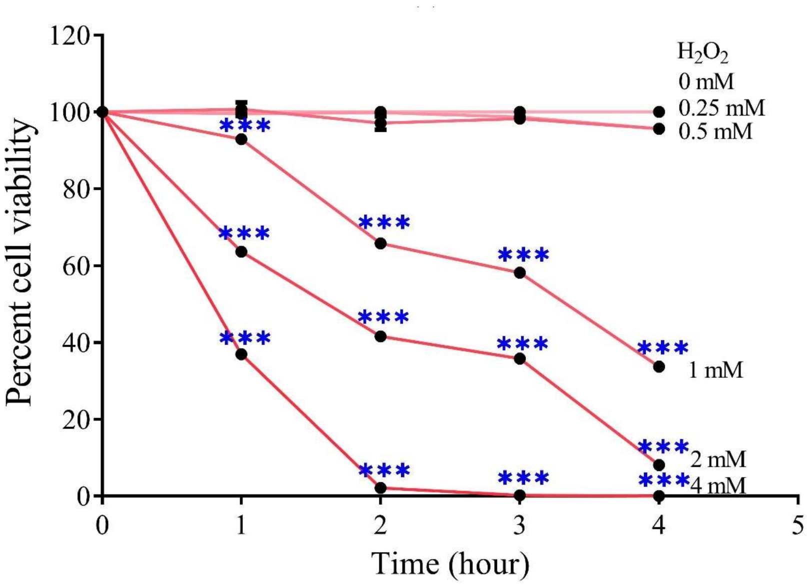

Figure S3 Survival curve of fibroblasts challenged with H_2O_2 (0-4 mM) at time-clause for 1-4 h.

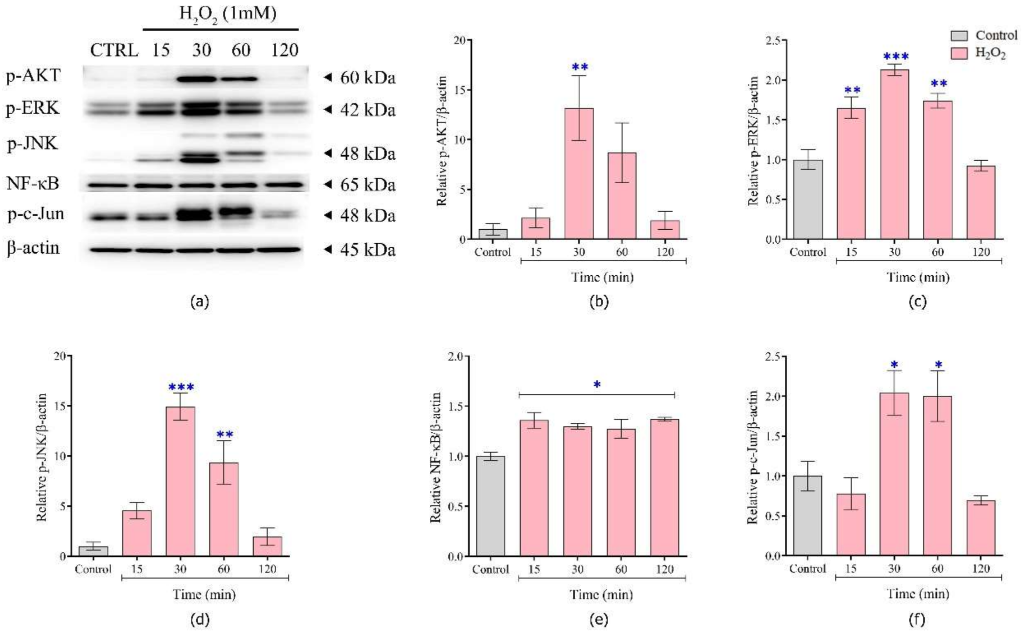

Figure S4 Time-course study of cell signaling changes in fibroblast cells exposed to H2O2. (a) Rep-resentative Western blot bands of each signaling protein. (b) p-AKT/β-actin. (c) p-ERK/β-actin. (d) p-JNK/β-actin. (e) NF-*κ*B/β-actin. (f) p-c-Jun/β-actin. Data are presented as mean ± SEM of $n \geq 3$. * $p < 0.05$, ** $p < 0.01$, and *** $p < 0.001$ when compared to the vehicle-treated group.

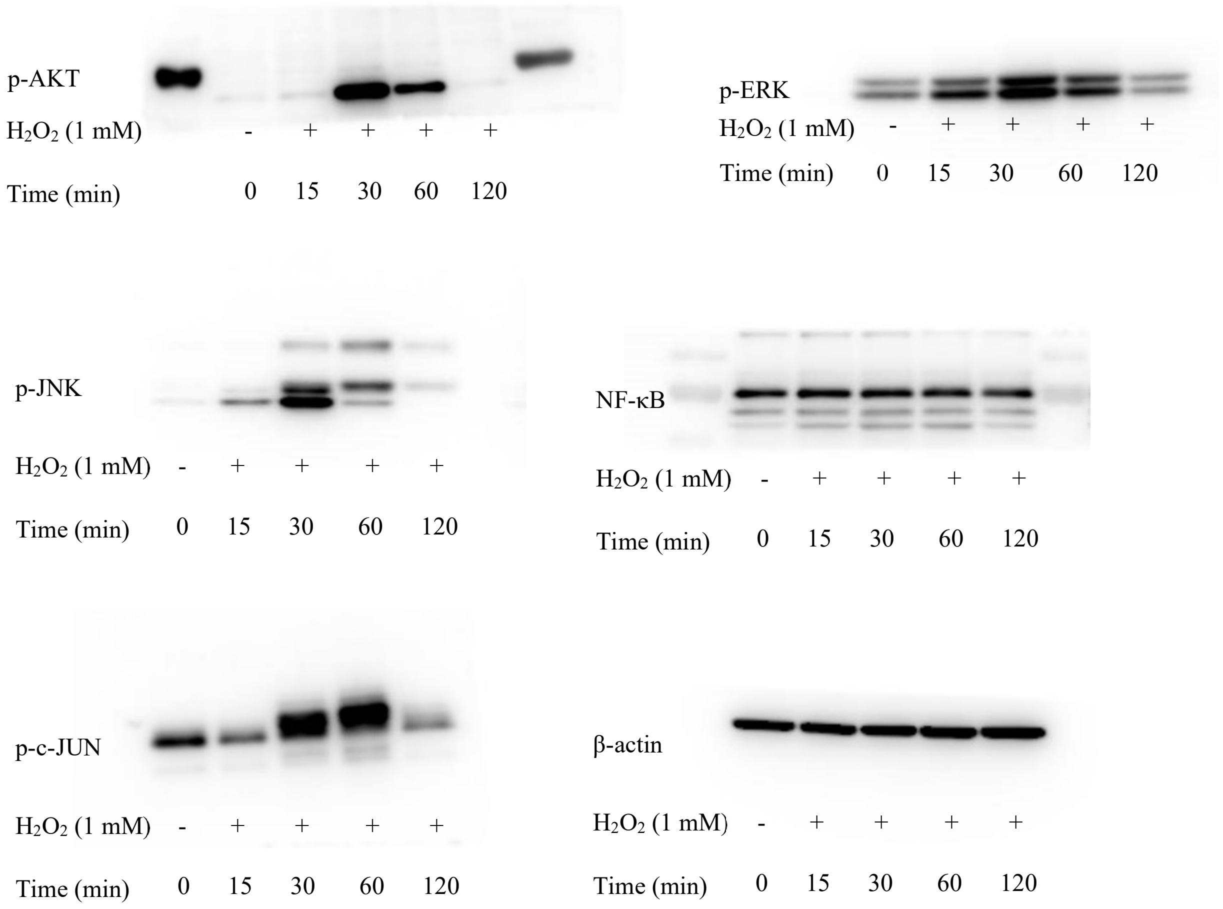

Figure S5 Original images of the Western blot band intensities shown in Figure S4

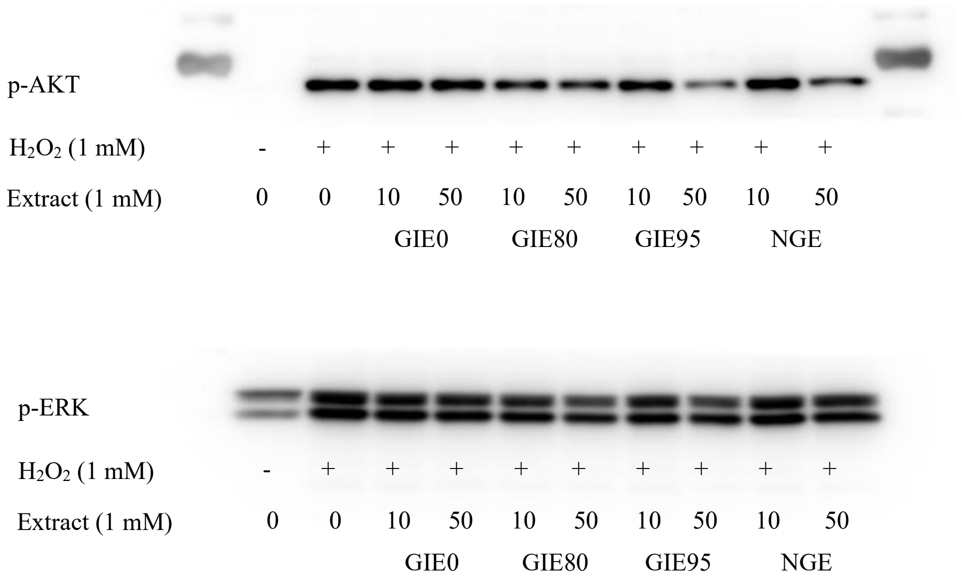

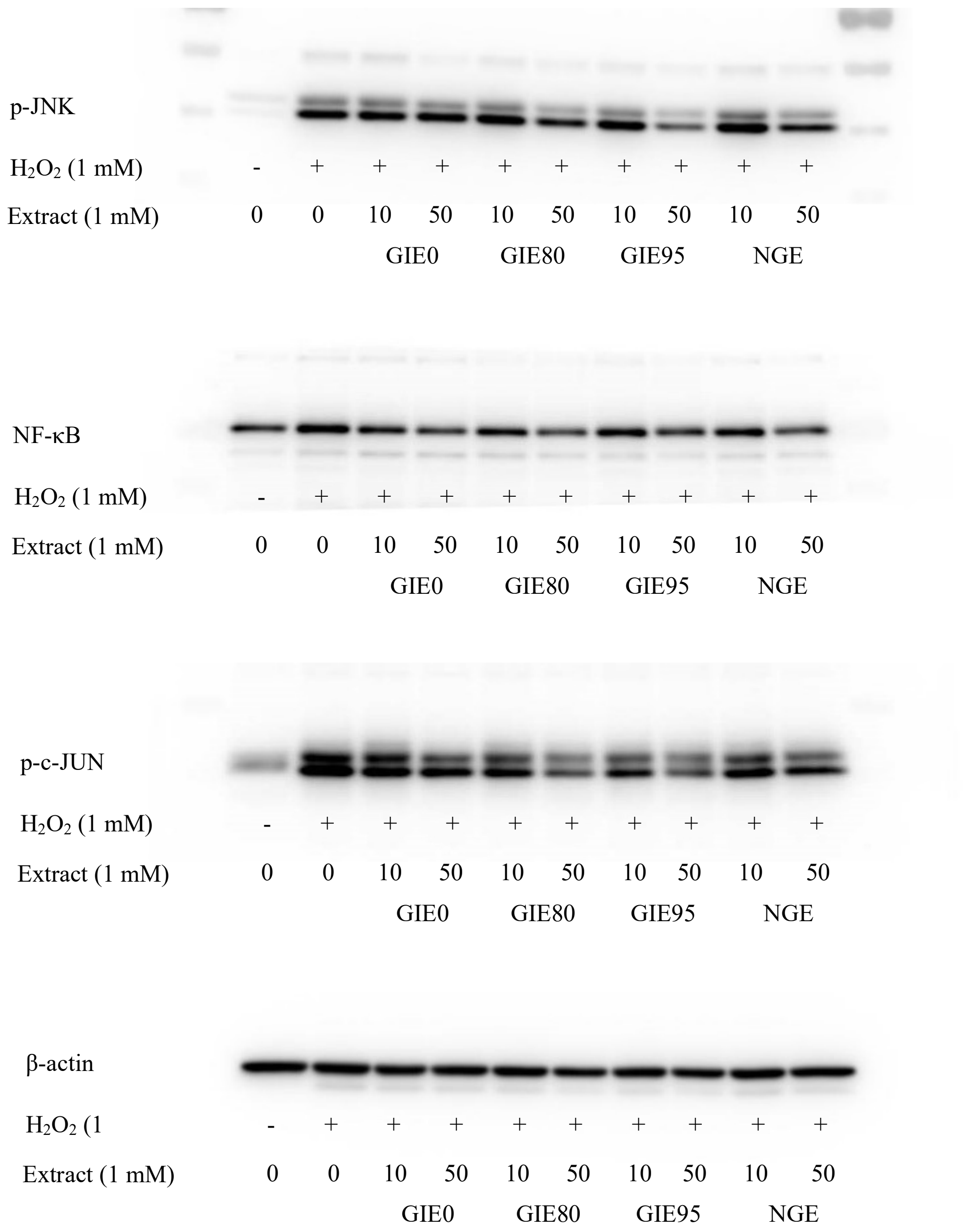

Figure S6 Original Western blot images corresponding to the band intensities presented in Figure 4.
